# Paroxysmal Sneezing

**Published:** 1895-08

**Authors:** W. Scott Renner

**Affiliations:** Buffalo N.Y., Professor of laryngology, Medical Department, Niagara University; Laryngologist to the Sisters of Charity Hospital, etc.


### PAROXYSMAL SNEEZING.

By W. SCOTT RENNER, M.D., C. M., Buffalo, N. Y.,

Professor of laryngology, Medical Department, Niagara University; Laryngologist to the Sisters of Charity Hospital, etc.

THIS title has been chosen in order to direct attention to a very common condition, without presupposing any theory for the causation or pathology of an association of symptoms, of which the chief and most distressing are paroxysms of sneezing, that in many cases occur daily and in some cases several times a day throughout the year.

Paroxysms of sneezing, associated with profuse watery discharge from the nose and eyes, swelling of the nasal mucous membrane and nasal obstruction, which may be transient, are described as reflex in character. Certain physiological reflex actions commonly occur as the result of the irritation of the healthy mucous membrane of the nose ; these are sneezing, lachrymation and coughing. For example, mechanical irritation of the anterior portion of the septum and turbinated bones in a case where the reflexes are of normal intensity may produce sneezing, lachrymation and rhinorrhea. If we pass the probe back along the septum or turbinated bones, or if we touch the pharynx near the Eustachian tubes with the probe, the nature of the reflex is changed and the patient coughs instead of sneezing.

These symptoms, sneezing, rhinorrhea, and the like, are found in several so-called diseases of the nasal mucous membrane. When occurring at certain seasons of the year, as early summer and early autumn, this group of symptoms are familiarly known as rose cold and hay-fever, and the pollen from the rose and certain grasses are supposed to be the exciting causes of the condition. There is another class of these cases, in which the attacks of sneezing do not limit themselves to any season of the year. In these the condition persists throughout the year, the attacks of sneezing coming on, perhaps, every day. These cases are named sneezing catarrh, false hay-fever, or other similar names. Cases of this last class are of such frequent occurrence that I wish to make a few general remarks concerning their symptoms, the local conditions in the nose associated with them, their relation to hay-fever and asthma, and the treatment which I have found most beneficial.

These patients present themselves with an account of their condition much as follows : They state that they have suffered from attacks of sneezing for years, occurring at intervals all the year round, are worse during the winter, or, perhaps, in the summer ; on getting out of bed in the morning, or at any time during the night, the patient is immediately attacked by a violent paroxysm of sneezing, which may last for two or three hours. Dust, smoke or the atmosphere of a close room, a warm theater or concert hall, is certain to bring on an attack which compels the patient to leave the room or be very much embarrassed by a fit of sneezing. Fatigue or worry may, in these cases, bring on an attack, and the discharge from the nose and eyes is profuse and like water. These attacks are accompanied by tickling of the whole of the anterior of the nose, sometimes reaching into the fauces. During the attack, the patient complains of oppression of the chest; there may be a history of asthma in the family; a strong mental impression will often arrest an attack. After this condition has persisted for years, the patient may be awakened in the night with an asthmatic paroxysm, accompanied by coughing, which will probably occur night after night at the same hour. After this state of affairs has lasted for some time, the patient becomes irritable and nervous, complains of frontal headache and has a wornout, haggard look, produced by the constant mouth-breathing and want of sleep.

A rhinoscopic examination of several of these cases will not reveal a similar condition in each. In some there is but little apparent change of the mucous membrane; in others there may be excessive hypertrophy of the tissue over the lower turbinated bones, or of that over both the lower and middle turbinated bones, or there may be nasal polypi. There will, probably, be more or less deviations of and outgrowths from the nasal septum. The mucous membrane over the turbinated bones and the nasal septum in many, as well as in cases of true hay-fever, has a peculiar ashen gray color, is wet with the exuding serum, and has a soggy appearance, as if soaked in water. In others the mucous membrane is of a dark red color, but is bathed in the same watery secretions as the first class. Mulhall, of St. Louis, has compared these two conditions to the vaso-motor disturbance seen in rage, one man's face being white while another's is livid. On examining the nasal mucous membrane with a probe, sensitive spots are found on the septum and turbinated bones, the touching of which produce violent attacks of sneezing. These spots are identical in character with those described by Sajous and J. N. Mackenzie as occurring in hay-fever. This, we can easily understand, is simply an exaggeration of the normal nasal reflex of the anterior part of the nose described above. The cough reflex is often likewise exaggerated when we have paroxysms of coughing at night accompanied by asthmatic breathing.

A person who has not passed through an experience of this kind cannot appreciate how mortifying it is to be compelled to sneeze perhaps every morning for years, to feel that an attack may come on at any moment and always at times when it will most embarrass and mortify the sufferer; for example, in the theater, during a call or at the dinner table. Some patients are often compelled to use a handkerchief constantly for hours daily, especially in the morning, to receive the flow of serum and mucus. The hay-fever patient is fortunate compared with the individual suffering from the more chronic disease, for his trouble lasts but a few weeks, while hay-fever resorts are abundant and easily reached; but the poor sneezer must go on suffering the year round, unless treatment affords him relief. I have met with sneezing catarrh more frequently than hay-fever, my attention being first directed to this disorder because I suffered from it myself. I shall not attempt, at present, to report any of the cases of this condition which have come under my notice, but will call attention to certain points in them which illustrate their relation to hay-fever and asthma.

Although there are many of these cases in which the patients are not affected by the pollen of the plants which excite hay-fever and rose cold, nevertheless there are others that are much worse during the hay-fever season; while, on the other hand, many patients who have suffered from hay-fever for years, begin after a time to suffer from a similar condition all the year round identical in character with the condition which I have described.

Likewise, many cases of bronchial asthma are accompanied by paroxysmal sneezing, and many of those suffering from paroxysmal sneezing, after years, develop bronchial asthma, like their brethren with hay-fever. Ringer recognised, years ago, the intimate relation of these three,—paroxysmal sneezing, hay-fever and asthma. He gives an exhaustive description of these conditions in earlier editions of his work on therapeutics, and illustrates their intimate relation to each other by the citation of cases.

It is evident from the clinical history of cases of hay-fever, rose cold and sneezing catarrh that they are identical in character, only differing in the exciting cause which brings on an attack and in their duration. Hay-fever and rose cold are excited by the pollen of certain flowers and plants and, consequently, only occur when the pollen of these flowers and plants are found in the atmosphere. In the other cases the attacks are brought on by a variety of causes, such as contact of the feet with a cold floor in the morning, the atmosphere of a warm room, and the like; and as such causes are always present when the local conditions of the mucous membrane are susceptible to irritation, they are likely to continue throughout the year unless successfully treated.

I do not wish, in this paper, to describe the pathology of these conditions, but if we accept the theory that asthma and hayfever are due to a vaso-motor disturbance, and call hay-fever rhinitis vaso-motorea periodica, we must give to this condition the name rhinitis vaso-motorea chronica, and so on. Whatever theory is offered, the one must be accepted if the other is.

The same distinction is found here as between hay-asthma and bronchial asthma due to other causes. I should, however, prefer the name coryza vaso-motorea to rhinitis vaso-motorea, because there is really, in these cases, no inflammation, which is essentially a part of the disease and, if present, is only accidental. The cases which have just been described must not be confounded with that condition which has been called nasal rhinorrhea, in which there is a constant and profuse running of serum from the nose, perhaps for hours daily. Several of these cases, by different authors, have been cited by Bosworth in his book. I have met this condition but twice in my experience. I am inclined to believe that the constitutional symptoms are due to the local trouble, *i. e.*, mouth-breathing and want of sleep from nasal obstruction, but not so much to a neurotic habit or gouty or lithemic diathesis, although I believe that diet and exercise will do much to hasten a cure. The neurotic habit and psychical condition has probably much more to do with the production of true hay-fever than with causing paroxysmal sneezing, although the mucous membrane itself is more susceptible to external influences in these cases.

### TREATMENT.

The special diseased conditions of the nasal mucous membrane which predispose to paroxysms of sneezing, as has already been stated, are the same as those which predispose to hay-fever and rose cold, the chief of which are hypertrophic rhinitis, deflections and outgrowths of the nasal septum and nasal polypi, or any other obstructive conditions of the nose, which tend to produce chronic turgescence of the blood-vessels of the mucous membrane. Therefore, special indications for treatment are found by a careful investigation and diagnosis of each case. Whatever lesion is found should be treated in the same manner in which it would be treated were there no such trouble present as the paroxysmal sneezing. Hypertrophies, deflections of the septum and nasal polypi should be removed by the most approved surgical methods. In most of these cases there is, in addition to the gross lesions or, perhaps, without the gross lesions, the hypersensitive spots on the mucous membrane described above. These, of course, require additional treatment before the unpleasant symptoms, for which the patient has consulted you, disappear. I treat these, when on the turbinated bones and on the septum,—and they occur most frequently on the septum,—by touching them with pure carbolic acid, or with a solution of chromic acid in water of a 4 per cent. to 10 per cent. strength. This should, of course, be repeated until all the sensitive spots are made out and cauterized. This method of touching the spots I should prefer to the use of the galvano-cautery, for I do not think that it is possible to use the cautery to any extent without producing a cicatrix. It seems to me all that is needful is counter-irritation, which is produced by the use of the acids of which I speak. This treatment, of course, should be supplemented, in most cases, by constitutional measures. I generally administer drop doses of Fowler's solution after meals, for small doses of arsenic seem to act well in controling the sneezing; at the same time I advise light exercise in the open air and sunlight. The patient should avoid stimulating diet and the use of tobacco, especially cigarettes.

As soon as all pathological conditions of the nasal mucous membrane have been removed, and all sensitive areas producing either sneezing or coughing have been carefully and thoroughly cauterized, the reflex manifestations disappear, perhaps only for a time, yet often permanently. Should they return, it will only be in a modified and mild form, when the spots which have been overlooked at the first treatment should be sought out and cauterized. Perseverance in this sort of treatment will practically cure most cases and relieve all. This is the same treatment which I follow locally in the treatment of hay-fever. I have been led to conclude, from cases that I have seen, that hay-fever, rose cold and sneezing catarrh are identical, the only difference being in the exciting cause of the paroxysm. The reasons which have led me to reach this conclusion are that the character of the attacks are the same, the appearance of the mucous membrane and the associated pathological conditions identical, while the same forms of treatment are the most beneficial in the majority of cases.

We must not, however, forget that in all cases of chronic nasal catarrh the nose is susceptible to the slightest irritation. These patients are said to be always taking cold; this cold taking varies in frequency with the amount of pathological change in the nasal mucous membrane. These attacks of cold in the head are often a passing paroxysm of sneezing; it seems to me that the difference between these cases and those of the classes of which I have spoken is more one of degree than of character. In many cases, whether of hay-fever, rose cold or sneezing catarrh, psychical influence seems to have considerable to do with precipitating an attack. An attack of hay-fever, for instance, which should have come on according to the patient's reckoning on August 20th, at 4 P. M., can be delayed a couple of hours by turning back the clock that much. J. N. Mackenzie produced an attack of rose cold in a susceptible lady by suddenly producing an artificial rose in front of her. Dread of an attack of sneezing has often brought such an attack about in cases of sneezing catarrh.

361 PEARL STREET.

